# Adipose tissue inflammation and *VDR* expression and methylation in colorectal cancer

**DOI:** 10.1186/s13148-018-0493-0

**Published:** 2018-04-25

**Authors:** Daniel Castellano-Castillo, Sonsoles Morcillo, Mercedes Clemente-Postigo, Ana Belén Crujeiras, Jose Carlos Fernandez-García, Esperanza Torres, Francisco José Tinahones, Manuel Macias-Gonzalez

**Affiliations:** 1 1Unidad de Gestión Clínica de Endocrinología y Nutrición del Hospital Virgen de la Victoria, Instituto de Investigación Biomédica de Málaga (IBIMA), Universidad de Málaga, Málaga, Spain; 2 CIBER Fisiopatología de la Obesidad y Nutrición (CB06/03), Madrid, Spain; 3 Laboratory of Molecular and Cellular Endocrinology, Instituto de Investigación Sanitaria (IDIS), Complejo Hospitalario Universitario de Santiago (CHUS/SERGAS), Santiago de Compostela University (USC), Santiago de Compostela, Spain; 4 CIBER Fisiopatología de la Obesidad y la Nutrición (CIBERobn), Madrid, Spain; 5 Unidad de Gestión Clínica de Oncología Intercentros Hospital Universitario Virgen de la Victoria, Málaga, Spain

**Keywords:** Vitamin D, *VDR*, DNA methylation, Low-grade inflammation, Colorectal cancer, Adipose tissue

## Abstract

**Background:**

Lack of vitamin D (VD) has been associated with colorectal cancer (CRC). VD has anti-inflammatory effects and regulates several cellular pathways by means of its receptor, including epigenetic modifications. Adipose tissue dysfunction has been related to low-grade inflammation, which is related to diseases like cancer. The aim of this study was to explore the relationship between serum 25-hydroxyvitamin D (25(OH)D), adipose tissue gene expression of VD receptor (VDR), pro-inflammatory markers, and the epigenetic factor DNA methyltransferase 3a (DNMT3A) as well as VDR promoter methylation in CRC.

**Methods:**

Blood and visceral adipose tissue from 57 CRC and 50 healthy control subjects were collected. CRC subjects had lower serum 25(OH)D levels and higher VDR gene expression, and these were negatively correlated in the CRC group.

**Results:**

Adipose tissue *NFκB1*, *IL6*, and *IL1B* gene expression were higher in the CRC subjects than in the control subjects. 25(OH)D correlated negatively with *NFκB1* and CRP. In turn, CRP correlated positively with *NFκB1*, *IL6*, *IL1B*, and *VDR* gene expression as well as *NFκB1* that correlated positively with *IL6* and *IL1B*. *DNMT3A* mRNA was negatively correlated with serum 25(OH)D and positively correlated with *VDR* DNA methylation. *VDR* DNA methylation at position 4 had lower levels in the CRC group. Global *NFκB1* methylation at dinucleotide 3 was lower in the CRC group.

**Conclusion:**

Our results suggest that adipose tissue may be a key factor in CRC development. The low 25(OH)D levels and high adipose tissue *VDR* expression in CRC may, at least in part, mediate this relationship by modifying adipose tissue DNA methylation and promoting inflammation.

**Electronic supplementary material:**

The online version of this article (10.1186/s13148-018-0493-0) contains supplementary material, which is available to authorized users.

## Background

Colorectal cancer (CRC) has become one of the most important health issues of our time due to its elevated prevalence, increasing incidence, morbidity, associated costs, and mortality rates. Several risk factors have been linked to CRC development: population aging, lack of physical activity, obesity, low fruit and vegetable intake, tobacco use, alcohol consumption, and other unknown factors [1].

Vitamin D (VD) can be incorporated from diet (vitamin D_2_ or D_3_) or synthetized by photoactivation of 7-dehydrocholesterol to previtamin D_3_ in the skin, a process that is mediated by sunlight [2]. Then, vitamin D_2_/D_3_ is converted to 25-hydroxyvitamin D (25(OH)D) by CYP2R1 in the liver, which is the main form of plasma vitamin D. The conversion of 25(OH)D to the active form, the 1,25-hydroxyvitamin D (1,25(OH)D), is carried out by the enzyme CYP24A1 in the kidneys [2]. Vitamin D functions primarily through the VD receptor (VDR), which is regulated by environment, genetics, and epigenetics [2].

Several studies have related plasma 25-hydroxyvitamin D (25(OH)D) levels with CRC [3–5], finding that low circulating 25(OH)D levels have been associated with CRC [4], and high 25(OH)D levels correlate with low risk for the onset of CRC [5]. A number of studies have reported the benefits of VD in processes such as metabolic modulation, autoimmunity, cardiovascular function, and cancer [6]. In fact, it has been proposed that calcitriol has anti-tumor effects [7, 8] as well as a direct effect on tumor development by acting as a tumor repressor in many solid tumors including CRC [9]. In a clinical trial carried out with patients who suffered from colorectal adenoma, the administration of VD together with calcium was able to reduce the expression of genes implicated in CRC development [10].

A number of studies have noted the relationship between CRC and low-grade inflammation [11]. Specifically, CRC patients have dysfunctional adipose tissue that might be a key contributor to the inflammatory state, by the secretion of several detrimental molecules such as tumor necrosis factor alpha (*TNFA*), interleukin-6 (*IL6*), and nuclear factor κ-light-chain-enhancer of activated B cells 1 (*NFκB1*) [12]. Adipose tissue has traditionally only been considered as an energy storage organ. Nevertheless, the importance of this tissue in systemic physiology and especially in systemic inflammation has been pointed out in recent years [13]. Adipose tissue expresses proteins related to VD metabolism [14], and it has been proposed that it can act as VD storage tissue [15]. The active form of VD, 1,25-dihydroxyvitamin D3 (1,25(OH)_2_D_3_), is able to modify adipocyte and adipose tissue physiology via the VDR [16, 17], decreasing the expression of pro-inflammatory cytokines in adipose tissue [18]. Therefore, VD might be a key factor for the higher risk of CRC in obese subjects, since low serum 25(OH)D levels or impaired adipose tissue responsiveness to VD might lead to a higher inflammatory state, which is directly implied in cancer development. However, the precise mechanism by which VD leads to a decrease in inflammation is not completely clear.

DNA methylation is an epigenetic regulatory process in which cytosine residues are methylated normally within CpG dinucleotides, referred to as CpG, and it is usually associated with gene repression, although it has also been related to gene activation in some cases [2, 19, 20]. Epigenetic mechanisms are susceptible to environmental factors such as diet, exercise, smoking, and hormones, and could be the basis for factors associated with an increased risk of cancer development such as obesity, inflammation, diabetes, or metabolic syndrome, as well as tumor onset or development [21–26]. DNA methylation is carried out by DNA methyltransferases *DNMT1*, *DNMT3A*, and *DNMT3B*, being *DNMT1* implied in the maintenance of DNA methylation and *DNMT3A* and *DNMT3B* in de novo DNA methylation processes. Furthermore, the relationship between vitamin D and DNA methylation has been analyzed in several studies, and 25(OH)D levels appear to control DNA methylation or demethylation [27], a mechanism in which DNA methyltransferases could be involved [28]. Furthermore, adipose tissue *DNMT3A* overexpression provokes a rise in adipose tissue inflammation in mice, which could be involved in adipose tissue-related diseases and CRC [29].

Therefore, we hypothesized that dysfunctional adipose tissue may play a major role in CRC development. 25(OH)D could be involved in epigenetic changes in adipose tissue triggering a change in the inflammatory profile, which could promote CRC onset and/or development. Thus, the aim of this study was to test serum levels of 25(OH)D, as well as the gene expression of the epigenetic factor *DNMT3A* and inflammatory markers in adipose tissue in CRC. We also studied *VDR* and *NFκB1* DNA methylation in adipose tissue to determine the possible role of the VD system in the epigenetic regulation of VDR and *NFκB1* gene.

## Methods

### Subjects

A total of 57 participants with CRC who underwent colorectal surgery and 50 control subjects who underwent hiatal hernia surgery or cholecystectomy were recruited from the Virgen de la Victoria University Hospital (Málaga, Spain) during 2012–2013.

Patients were excluded if they had cardiovascular disease, arthritis, acute inflammatory disease, infectious disease, and renal disease, and were receiving drugs that could alter the lipid or glucose profile, were undergoing treatment with calcium or vitamin D supplements, or if they consumed > 20 g ethanol per day at the time of inclusion in the study. The study was conducted in accordance with the guidelines laid down in the Declaration of Helsinki. All participants gave their written informed consent (0311/PI7), and the study was reviewed and approved by the Ethics and Research Committee of Virgen de la Victoria Hospital.

Epiploic visceral adipose (VAT) tissue was obtained during surgery, washed in physiological saline solution, and immediately frozen in liquid nitrogen. Biopsy samples were maintained at − 80 °C until analysis.

### Laboratory measurements

Before surgery and after an overnight fast, blood samples were obtained from the antecubital vein and placed in vacutainer tubes (BD vacutainer™). The serum was separated by centrifugation for 15 min at 4000 rpm and immediately frozen at − 80 °C until analysis. Serum glucose, cholesterol, triglycerides, HDL cholesterol (HDL-C), and C-reactive protein (CRP) were measured in a Dimension autoanalyzer (Dade Behring Inc.) by enzymatic methods (Randox Laboratories Ltd.). LDL cholesterol (LDL-C) was calculated using the Friedewald equation. Insulin was quantified by radioimmunoassay supplied by BioSource International Inc., Camarillo, CA, USA. The homeostasis model assessment of insulin resistance (HOMA-IR) was calculated with the following equation: HOMA-IR = fasting insulin (μIU/mL) × fasting glucose (mmol/L)/22.5. Serum 25(OH)D and parathyroid hormone levels were determined by enzyme immunoassay (ELISA) kits (Immundiagnostik and DRG Diagnostics, respectively). Corrected calcium was calculated using the following equation: fasting calcium (mg/dl) + 0.8 × (4-fasting albumin (g/dl)).

### Visceral adipose tissue RNA isolation and real-time quantitative PCR

Total RNA isolation from VAT was obtained using RNeasy Lipid Tissue Mini Kit (Qiagen GmbH, Hilden, Germany). The purity of the RNA was determined by the 260/280 absorbance ratio on the NanoDrop. The integrity of the total purified RNA was checked by denaturing agarose gel electrophoresis and ethidium bromide staining. For first strand cDNA synthesis, a constant amount of 1 μg of total RNA was reverse transcribed using random hexamers as primers and Transcriptor Reverse Transcriptase (Roche, Mannheim, Germany). Gene expression was assessed by real-time PCR using an Applied Biosystems 7500 Fast Real-Time PCR System (Applied Biosystems, Darmstadt, Germany) with TaqMan technology as previously described [30]. The commercially available and prevalidated TaqMan primer/probe sets used were as follows: *VDR* (Hs01045840_m1, RefSeq. NM_000376.2, NM_001017535.1 and NM_001017536.1), *NFκB1* (Hs00765730_m1, RefSeq. NM_001165412.1 and NM_003998.3), *DNMT3A* (NM_001320893.1, NM_022552.4, NM_153759.3, NM_175629.2), *IL6* (Hs00174131_m1; RefSeq. NM_000600.4, NM_001318095.1), *IL1B* (Hs00174097_m1; NM_000576.2), and *PPIA* (4326316E, RefSeq. NM_021130.3), used as endogenous control for the target gene in each reaction.

### Protein extraction and western blot

For total protein extract preparation, adipose tissue samples were washed once in erythrocyte lysis buffer (sacarose 320 mM, Tris-HCl pH 7.5 10 mM, MgCl_2_ 5 mM, Tritonx-100 1%) and PBS for 20 min at 4° with agitation. Samples were homogenized using T-PER tissue protein extraction reagent (Thermofisher, USA) and Ultra Turrax Homogenizator and then centrifuged to discard the pellet and the upper fatty layer. Samples were resolved by SDS-PAGE and transferred to a nitrocellulose membrane to perform the Western blotting. A mouse monoclonal anti-VDR (sc 13133, Santa Cruz Biotechnology) was used as primary antibody and a goat anti-mouse IgG-HRP (sc-2005, Santa Cruz Biotechnology) as secondary antibody. Clarity Western ECL substrate (Bio-Rad, USA) was used for detection, and the target protein was determined by using the total protein determined by ponceau staining to normalize the quantification. All experiments were performed in duplicate.

### Pyrosequencing

The DNA methylation status was determined by pyrosequencing using the PyroMarkTMQ96 ID Pyrosequencing System (Qiagen). We used a premade Pyromark CpG assay for VDR (PM00051443) and *NFκB1* (PM00110908). An overview of the analyzed regions for VDR and *NFκB1* is depicted in Supplementary Additional files 1 and 2: Figures S1 and S2, respectively. Briefly, NFκB1 is located at chromosome 4. The region 103,423,139-103,423,177 (39 bp length) was analyzed, which contains more than 30 transcription factor binding sites (TFBS) determined by ChiP experiments by the ENCODE project [31]. Besides, the assay was located inside additional TFBS according to the Open Regulatory Annotation database (ORegAnno) [32], as SMARCA4, SPL1, STAT1, RBL2, RB1, and ETS1. VDR is located at chromosome 12. The region 48,299,419-48,299,455 (37 bp length) was analyzed, which contains several TFBS determined by ChiP experiments by the ENCODE Project [31], as POLR2A, ATF2, CTCF, EZH2, E2F6, GATA2, GATA3, CEBPB, or POL2. Additional TFBS are present according to ORegAnno [32], as SMARCA4, SPL1, STAT1, RBL2, RB1, and ETS1. DNA methylation analyses were performed using bisulfite-treated DNA followed by a highly quantitative analysis based on PCR-based pyrosequencing. The bisulfite conversion was conducted with 2-μg genomic DNA isolated from VAT using Qiazol (Qiagen) and 0.1 μM citrate ethanol solution. Then, the PCR was performed in a total volume of 25 μL, with a final primer concentration of 0.2 μM. One of the primers was biotinylated in order to purify the final PCR product using Sepharose beads. The biotinylated PCR amplification was purified using the Pyrosequencing Vacuum Prep Tool (Qiagen). Finally, 15 μL of the PCR products was pyrosequenced using the PyroMarkTMQ96 ID Pyrosequencing System, using a 0.4-μM sequencing primer.

The methylation level was expressed as the percentage of methylated cytosine over the sum of methylated and unmethylated cytosines. Non-CpG cytosine residues were used as built-in controls to verify bisulfite conversion. The values are expressed as the mean for all the sites and individually for six CpGs at the *VDR* gene promoter and seven CpGs at the *NFκB1* promoter. We also included unmethylated and methylated DNA as controls in each run (New England Biolabs). Inter-assay precision (% CV) was < 2.5%; intra-assay (% CV) was < 1.0%.

### Statistical analysis

**Fig. 1:**
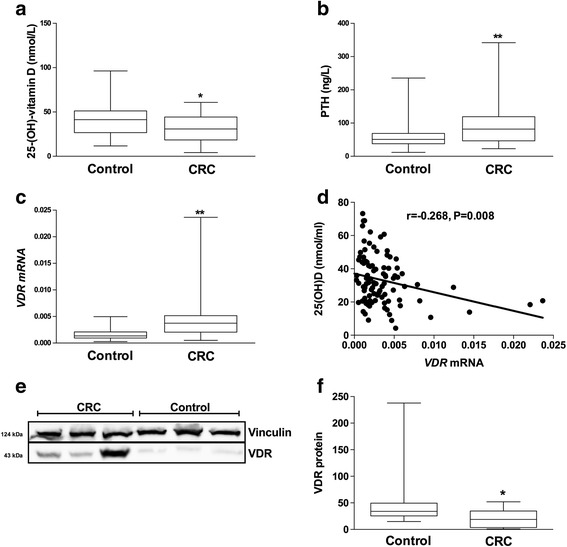
Comparison of serum 25(OH)D and PTH levels and adipose tissue VDR mRNA and protein in CRC patients and controls. Comparisons were performed using Student *t* test (for 25(OH)D) and Mann-Whitney *U* test (for serum PTH and adipose tissue *VDR* mRNA and VRD protein). Serum levels of **a** 25(OH)D, and **b** PTH was measured by ELISA in both the control and CRC group. **c** Adipose tissue VDR mRNA expression was measured by qPCR (*n* = 107), and Spearman’s correlation (**d**) between serum 25(OH)D and adipose tissue VDR mRNA in the whole study population was performed. Comparison of adipose tissue VDR protein (**e**, **f**) analyzed by Western blot (*n* = 18). * and ** mean *p* < 0.05 and *p* < 0.01, respectively. Parathyroid hormone (PTH), vitamin D receptor (VDR), colorectal cancer (CRC)

**Fig. 2:**
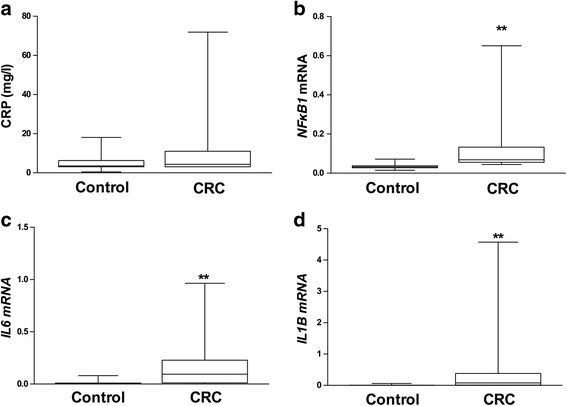
Serum and adipose tissue inflammatory markers. Comparisons were performed using non-parametric test (Mann-Whitney *U* test). Serum CRP levels (**a**) and adipose tissue *NFκB1* (**b**), *IL6* (**c**), and *IL1B* (**d**) gene expression in the control and CRC groups. ** means *p* < 0.01. C-reactive protein (CRP), nuclear factor kappa B subunit 1 (*NFκB1*), interleukin 6 (*IL6*), interleukin 1 beta (*IL1B*); colorectal cancer (CRC)

**Fig. 3:**
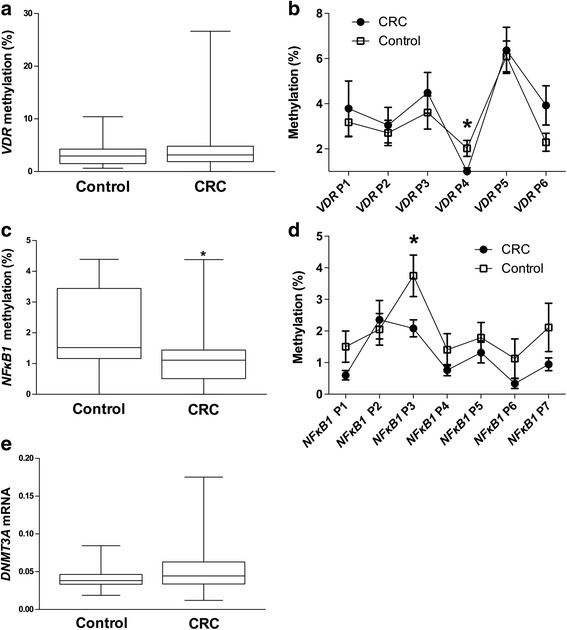
Methylation analyses at specific CpG dinucleotides for *VDR* and *NFκB1* promoters and gene expression of the epigenetic factor *DNMT3A* were performed to compare both the control and CRC groups. Comparisons of the global VDR methylation (**a**) and among the CpG dinucleotides analyzed (**b**) by Mann-Whitney *U* test. Non-parametric (Mann-Whitney *U* test) comparison for the global *NFκB1* methylation (**c**) and at specific CpG dinucleotides (**d**), as well as for the methyltransferase *DNMT3A* gene expression (**e**). * means *p* < 0.05. Vitamin D receptor (VDR), DNA methyltransferase 3a (*DNMT3A*), colorectal cancer (CRC)

The results are given as the mean ± SD (Table 1) and as a box plot with the minimum and maximum value (control case comparisons shown in Figs. 1, 2, and 3). Student’s *t* test was used for comparisons of the anthropometric and biochemical characteristics as well as serum 25(OH)D levels between the CRC and control groups. Mann-Whitney *U* test was performed for comparisons of serum CRP and PTH levels as well as for *VDR* methylation, mRNA, and protein expression levels and for *NFκB1* methylation and mRNA levels between the CRC and the control groups. Spearman’s correlation analyses were performed to study the correlations between 25(OH)D and *VDR* mRNA and to study the correlations for *NFκB1* DNA methylation. Partial correlation analyses corrected by gender were used to study the correlation among the study gene expressions and plasma levels of 25(OH)D and CRP. For the analysis, non-normal distribution variables were log-transformed. The analyses were performed with SPSS (Version 15.0 for Windows; SPSS Iberica, Spain). Values were considered to be statistically significant when *p* < 0.05.

**Table 1:** Anthropometric and biochemical variables of the study groups

	Control (*n* = 57)	CRC (*n* = 50)
Age (years)	64.94 ± 8.84	68.035 ± 8.43
Male/Female (%)*	68/32	45/55
BMI (kg/m^2^)	28.51 ± 4.21	27.61 ± 3.91
Waist (cm)	96.55 ± 11.64	97 ± 12.74
Glucose (mg/dl)	111.72 ± 28.77	125.035 ± 46.87
Insulin (μUI/ml)**	11.638 ± 6.54	6.23 ± 5.19
Triglycerides (mg/dl)*	142.3 ± 70.69	172.821 ± 87.54
Cho (mg/dl)**	220.68 ± 39.84	169.625 ± 43.57
HDL-C (mg/dl)**	53.28 ± 14.4	40.053 ± 15.12
LDL-C (mg/dl)**	136.61 ± 29.80	101.58 ± 35.67
Corrected calcium (mg/dl)**	8.99 ± 0.44	9.67 ± 0.65
Alkaline phosphatase (U/L)	72.67 ± 21.63	64.66 ± 22.81

*CRC* Colorectal Cancer Group, *BMI* Body Mass Index, *DM* Diabetes Mellitus, *Cho* Total Cholesterol, *HDL-C* High Density Lipoprotein Cholesterol, *LDL-C* Low density Lipoprotein Cholesterol

Results are presented as means ± S.D. *p < 0.05 CRC vs. Control; **p < 0.01 CRC vs. control according to t student’s test and Chi squared test for variables expressed as percentage

## Results

### Anthropometric and biochemical variables

Table 1 shows the biochemical and anthropometric characteristics of the study groups. There were no differences in age, BMI, or gender between the control and CRC groups. The CRC group had lower levels of insulin, total cholesterol, HDL-C, and LDL-C than the control group. In contrast, the CRC group presented higher levels of plasma triglycerides when compared with the control group.

### Serum 25(OH)D levels and adipose tissue VDR gene and protein expression

Our results showed that 12% in the control group and 26% in the CRC group were vitamin D deficient (25(OH)D lower than 20 nmol/L) according to the Endocrine Society Clinical Practice Guideline [33], although no significant differences were found according to Fisher’s test. Serum 25(OH)D levels were significantly lower in the CRC group than in the control group (Fig. 1a), while parathyroid hormone levels showed an inverse result (Fig. 1b). Contrary to serum 25(OH)D levels, adipose tissue *VDR* mRNA levels were higher in the CRC group than in the control group (Fig. 1c) which in turn correlated negatively with 25(OH)D (*r* = − 0.268; *p* = 0.008) (Fig. 1d). This correlation was maintained using a partial correlation analysis corrected by gender (*r* = − 0.273, *p* = 0.01). Accordingly, mRNA levels were translated to higher VDR protein levels in the CRC group with regard to the control group (Fig. 1e, f).

### Inflammatory status and relationship with circulating vitamin D

We checked the systemic inflammatory status by measuring serum CRP, as well as the inflammatory status of adipose tissue by determining mRNA levels of *NFκB1*, *IL6*, and *IL1B* gene expression. We found higher levels of adipose tissue *NFκB1*, *IL6*, and *IL1B* mRNA levels (Fig. 2b–d) in the CRC group with regard to the control group.

25(OH)D correlated negatively with adipose tissue NFκB1 mRNA (Table 2). Concordantly, we observed that serum CRP levels were negatively correlated with serum 25(OH)D levels and positively correlated with both VDR and *NFκB1* gene expression in adipose tissue (Table 3A). In turn, there was a positive correlation between *NFκB1* mRNA and both *IL6* and *IL1B* mRNA levels (Table 3B). Furthermore, a positive correlation was found between *NFκB1* mRNA and *VDR* (Table 3B).

**Table 2:** Partial correlation between serum 25(OH)D and adipose tissue *NFκB1* mRNA, *IL6* mRNA and *IL1B* mRNA corrected by gender in the whole population

	25(OH)D	
	r	p
Log(*NFκB1* mRNA)	−0.232	0.041
Log(*IL6* mRNA)	−0.125	0.251
Log(*IL1B* mRNA)	−0.106	0.339

*25(OH)D* 25-hydroxy-vitamin D, *NFκB1* Nuclear Factor Kappa B subunit 1, *IL6* Interleukin 6, *IL1B* Interleukin 1 beta

**Table 3:** Partial correlations of C-reactive protein (A) with serum 25(OH)D, adipose tissue *VDR* mRNA, *NFκB1* mRNA, *IL6* mRNA, and *IL1B* mRNA corrected by gender. Partial correlations corrected by gender of adipose tissue *NFκB1* mRNA (B) with IL 6 mRNA, *IL1B* mRNA and *VDR* mRNA

A	Log(C-reactive protein)	
	r	p
Log(25(OH)D)	−0.270	0.011
Log(*VDR* mRNA)	0.219	0.049
Log(*NFκB1* mRNA)	0.284	0.016
Log(*IL6* mRNA)	0.245	0.029
Log(*IL1B* mRNA)	0.272	0.016
B	*NFκB1* mRNA	
	r	p
Log(*IL6* mRNA)	0.688	0.000
Log(*IL1B* mRNA)	0.778	0.000
Log(*VDR* mRNA)	0.761	0.000

*25(OH)D* 25-hydroxy-vitamin D, *VDR* vitamin D receptor, *NFκB1* Nuclear Factor Kappa B subunit 1, *IL6* Interleukin 6, *IL1B* Interleukin 1 beta

### *VDR* and *NFκB1* methylation and association between the epigenetic factor *DNMT3A* and 25(OH)D

The DNA methylation status of the *VDR* promoter was determined by pyrosequencing, but no differences between the control and CRC groups were found (Fig. 3a). When individual *VDR* CpG positions were compared, significant lower *VDR* methylation at position 4 (*VDR* P4) was found in the CRC group when compared with the control group (Fig. 3b). *NFκB1* global methylation was lower in the CRC group than in the control group. A comparative analysis at each *NFκB1* CpG analyzed showed that *NFκB1* at position 3 (*NFκB1* P3) presented a lower methylation level in CRC with regard to the control group. Moreover, a negative trend (*r* = − 0.252, *p* = 0.061) was observed between *NFκB1* mRNA levels and *NFκB1* P3 and between *VDR* mRNA and the global *NFκB1* methylation (*r* = − 0.228; *p* = 0.064). Additionally, a negative and significant correlation was found between *VDR* mRNA and *NFκB1* at position 3 (*NFκB1* P3) (*r* = − 0.296; *p* = 0.015) and at position 4 (*NFκB1* P4) (*r* = − 0.327; *p* = 0.007). Furthermore, we analyzed *DNMT3A* gene expression in adipose tissue, which was higher in the CRC group than in the control group but without getting statistic significance (Fig. 3e). We also found a negative correlation (corrected by gender) between the gene expression of the epigenetic factor *DNMT3A* and serum 25(OH)D levels (*r* = − 0.264, *p* = 0.013). There was a positive correlation between adipose tissue *DNMT3A* gene expression and adipose tissue *VDR* DNA methylation in a partial correlation corrected by gender (*r* = 0.256, *p* = 0.034). We also found a positive correlation (Spearman’s correlation) between *DNMT3A* mRNA and *NFκB1* mRNA (*r* = 0.279, *p* = 0.009).

## Discussion

In this study, we found lower levels of serum 25(OH)D and higher levels of CRP, which is in accordance with previous studies [34, 35]. In addition, to our knowledge, this is the first study which has aimed at analyzing the putative relationship between the VDR in adipose tissue and CRC, taking into consideration the anti-inflammatory role that has been attributed to VD [18, 36]. This is based on the hypothesis that the pro-inflammatory profile of adipose tissue could contribute to the systemic inflammation which has been described to be related to CRC [24]. According to this hypothesis, we found that, apart from lower plasma 25(OH)D levels, CRC patients had higher adipose tissue mRNA levels of pro-inflammatory mediators. Interestingly, we also found significant differences in *VDR* gene expression between the CRC and the control group, which suggest that in fact, VD may be mediating an anti-inflammatory role in the adipose tissue of CRC patients [37]. Our study shows that adipose tissue *DNMT3A* mRNA correlates negatively with 25(OH)D and positively with adipose tissue *VDR* and *NFκB1* methylation, suggesting that VD could be involved in epigenetic modifications in both genes in adipose tissue by mechanisms involving the DNA-methyltransferase *DNMT3A*. Interestingly, the VDR CpGs analyzed were located in the promoter region of the VDR gene (Additional file 1: Figure S1) and inside several TFBS as *POLR2A*, *ATF2*, *CTCF*, *EZH2*, *E2F6*, *GATA2*, *GATA3*, *CEBPB*, or *POL2*, which some of them has been related to DNA methyltransferases recruitment [38, 39]. Changes in adipose tissue methylation status could be related to its pro-inflammatory profile, as previous studies have described a positive relationship between *DNMT3A* and inflammation in a murine model [29] as we have described in our study population.

It has been found that circulating CRP is associated with a higher risk of CRC [40], and high levels of serum CRP have been related to low levels of 25(OH)D [10], which is in agreement with our results showing a negative correlation between CRP and 25(OH)D levels. Adipose tissue dysfunction might play a crucial role in the promotion of different diseases including insulin resistance, diabetes, and cancer [41, 42]. Several mechanisms have been proposed to explain this relationship, including an altered adipokine secretion profile [24]. Some of these alterations may provoke the development of a chronic low-grade inflammatory state due to the production and secretion of pro-inflammatory cytokines by adipose tissue [41, 43, 44], which has been regarded as a favorable environment for tumor development [24]. Recently, it has been proven that cytokines secreted by adipose tissue have a direct effect on tumor aggressiveness and cancer cell migration in prostate cancer [45], and on colon cancer in mice [46]. Interestingly, it has been described that calcitriol decreases the adipose tissue chronic pro-inflammatory status by downregulating pro-inflammatory cytokine production in a process in which *NFκB1* and *VDR* are involved [47]. Indeed, *NFκB* has been implied in cancer development [12]*.* Our results concur with this idea since CRC patients showed higher adipose tissue *NFκB1* gene expression in comparison with control subjects, and *NFκB1* transcription levels correlated positively to *VDR*, *IL6*, and *IL1B* mRNA levels, confirming the relationship between *VDR* and inflammation [47]. These facts support the idea that low circulating 25(OH)D promotes a chronic low-grade inflammatory state in adipose tissue, the effects of which could be the release of pro-inflammatory cytokines inducing or stimulating colon tumors.

Calcitriol action is mediated by its receptor, *VDR*. *VDR* has also been associated with CRC. Specifically, a higher tumor expression of *VDR* in CRC correlates with a better prognosis, and there is a direct relation between the tumor differentiation level and *VDR* gene expression level [48, 49]. However, to our knowledge, there are no previous studies analyzing the relationship between adipose tissue *VDR* gene expression and CRC. Here, we found that there were higher adipose tissue *VDR* mRNA and protein levels in the CRC group when compared with the control group. These high *VDR* levels in adipose tissue might be due to a compensatory mechanism in response to the low 25(OH)D levels in these subjects, and it could be a sign of VD insufficiency. Moreover, it has been reported that the inflammatory factor TNF can activate *VDR* gene expression [50]. So, a lack of 25(OH)D might lead to an inflammatory process which could subsequently promote *VDR* expression as we observed in our study. High *VDR* expression without its ligand, calcitriol, has been shown to have an opposite effect in gene expression regulation and could alter epigenetic marks [51, 52].

In addition, although previous studies described a relationship between methylation status and 25(OH)D [27], which agrees with our observation between *DNMT3A* and 25(OH)D levels, this phenomenon has not previously been analyzed in adipose tissue. Therefore, to our knowledge, the present study is the first one to report a negative association between 25(OH)D levels and adipose tissue *DNMT3A* gene expression. Concordantly, *DNMT3A* mRNA levels were positively correlated with global *VDR* promoter methylation. This could suggest an epigenetic effect in adipose tissue via VD action. Interestingly, when the methylation of key individual CpGs was analyzed, we found that methylation levels at *VDR* P4 were significantly lower in CRC than in the control subjects. However, it should also be taken into consideration that other mechanisms might be involved in the regulation of gene expressions such as histone modifications or microRNAs. Therefore, further studies will be necessary to clarify the relevance of *VDR* methylation in the regulation of its expression in adipose tissue as well as the consequences that these epigenetic changes could have in CRC. We also found a possible regulation of *NFκB1* gene expression through DNA methylation. Furthermore, we saw an association between *VDR* mRNA levels and *NFκB1* DNA methylation, which could agree with previous studies although a deeper approach would be necessary to clarify the possible relationship between *VDR* and the VD signaling with the epigenetic control in *NFκB1* gene expression.

## Conclusions

Our results suggest that adipose tissue may be a key factor in CRC development. The low 25(OH)D levels in CRC and high adipose tissue *VDR* expression may, at least in part, mediate this relationship by modifying adipose tissue DNA methylation and promoting inflammation. Although more studies are needed to discover the precise mediators and mechanisms that determine this relationship, the possible mediation of adipose tissue in CRC should be borne in mind to create new treatments and preventive strategies for CRC.

## Additional files

Additional file 1: **Figure S1.** *VDR* promoter overview generated by UCSC genome Browser (https://genome.ucsc.edu). The sequence analyzed is highlighted in light blue, showing that is in the promoter region of VDR and inside a CpG island and several transcription factor binding sites (*POLR2A*, *ATF2*, *CTCF*, *EZH2*, *E2F6*, *GATA2*, *GATA3*, *CEBPB* and *POL2*) all of them determined by experimental procedures (ENCODE project). (DOCX 188 kb)

Additional file 2: **Figure S2.** *NFκB1* promoter overview generated by UCSC genome Browser (https://genome.ucsc.edu). The sequence analyzed is highlighted in light blue, showing that is in the promoter region of *NFκB1* and inside a CpG island and several transcription factor binding sites (*SMARCA4*, *SPL1*, *STAT1*, *RBL2*, *RB1* and *ETS1*) all of them determined by experimental procedures (ENCODE project). (DOCX 341 kb)

## Abbreviations

25(OH)D: 25-Hydroxyvitamin D
CRC: Colorectal cancer
CRP: C-reactive protein
DNMT3A: DNA methyltransferase 3a
HOMA-IR: Homeostasis model assessment of insulin resistance
IL1: Interleukin-1
IL6: Interleukin-6
NFκB1: Nuclear factor κ-light-chain-enhancer of activated B cells
TNFA: Tumor necrosis factor alpha
VAT: Visceral adipose tissue
VD: Vitamin D
VDR: Vitamin D receptor
