# Supplementary material for: Adipose tissue inflammation and VDR expression and methylation in colorectal cancer
Source: Clin Epigenetics. 2018 Apr 25;10:60. doi: 10.1186/s13148-018-0493-0 (PMC5921388; doi:10.1186/s13148-018-0493-0)
Supplement: Supplementary file 1 — Figure S1. VDR promoter overview generated by UCSC genome Browser (https://genome.ucsc.edu). The sequence analyzed is highlighted in light blue, showing that is in the promoter region of VDR and inside a CpG island and several transcription factor binding sites (POLR2A, ATF2, CTCF, EZH2, E2F6, GATA2, GATA3, CEBPB and POL2) all of them determined by experimental procedures (ENCODE project). (DOCX 188 kb) [file 13148_2018_493_MOESM1_ESM.docx]

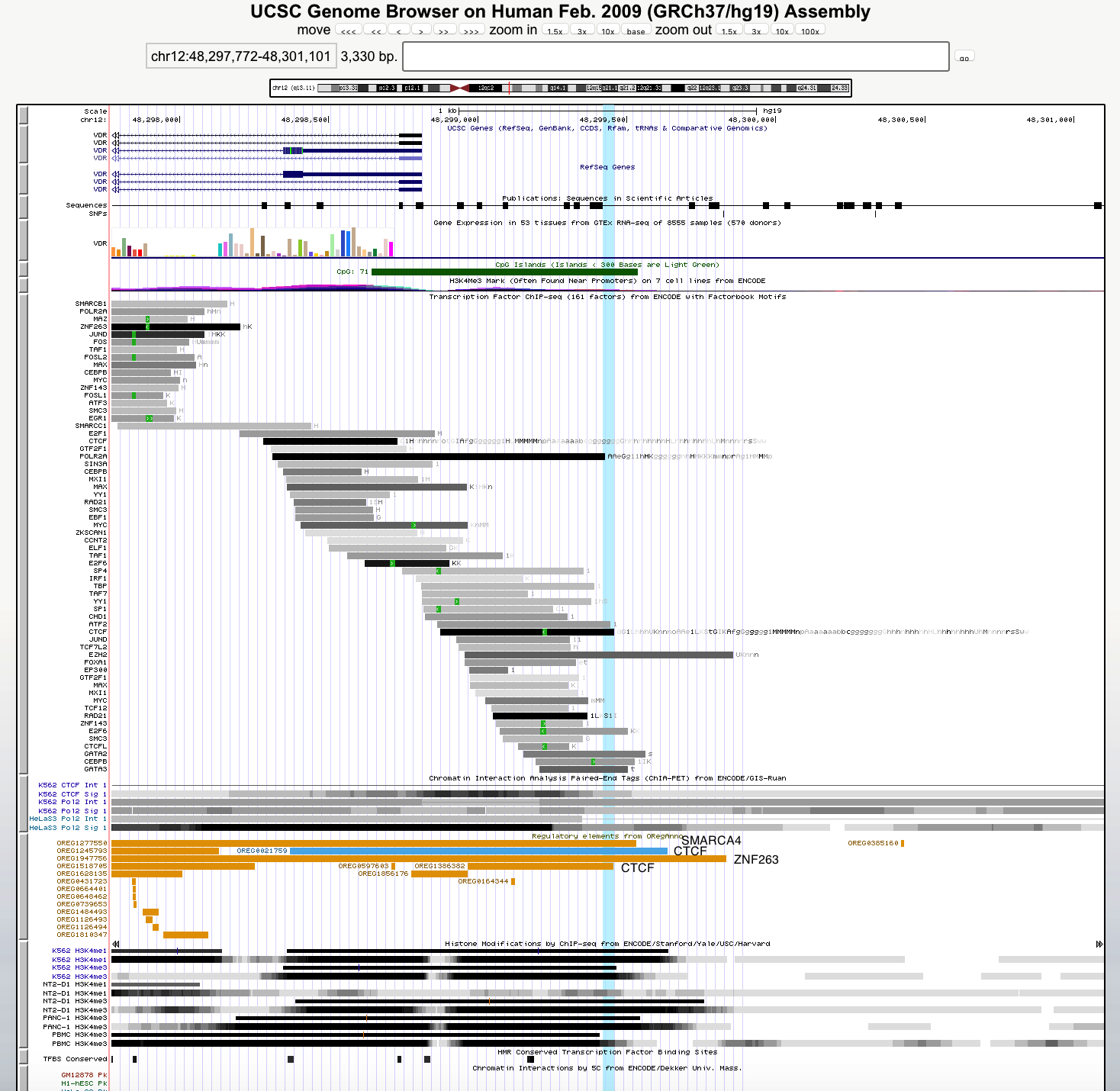


**Supplementary Figure 1.** *VDR* promoter overview generated by UCSC genome Browser (https://genome.ucsc.edu). The sequence analyzed is highlighted in light blue, showing that is in the promoter region of VDR and inside a CpG island and several transcription factor binding sites (*POLR2A*, *ATF2*, *CTCF*, *EZH2*, *E2F6*, *GATA2*, *GATA3*, *CEBPB* and *POL2*) all of them determined by experimental procedures (ENCODE project).
