# Supplementary material for: Adipose tissue inflammation and VDR expression and methylation in colorectal cancer
Source: Clin Epigenetics. 2018 Apr 25;10:60. doi: 10.1186/s13148-018-0493-0 (PMC5921388; doi:10.1186/s13148-018-0493-0)
Supplement: Supplementary file 2 — Figure S2. NFκB1 promoter overview generated by UCSC genome Browser (https://genome.ucsc.edu). The sequence analyzed is highlighted in light blue, showing that is in the promoter region of NFκB1 and inside a CpG island and several transcription factor binding sites (SMARCA4, SPL1, STAT1, RBL2, RB1 and ETS1) all of them determined by experimental procedures (ENCODE project). (DOCX 341 kb) [file 13148_2018_493_MOESM2_ESM.docx]

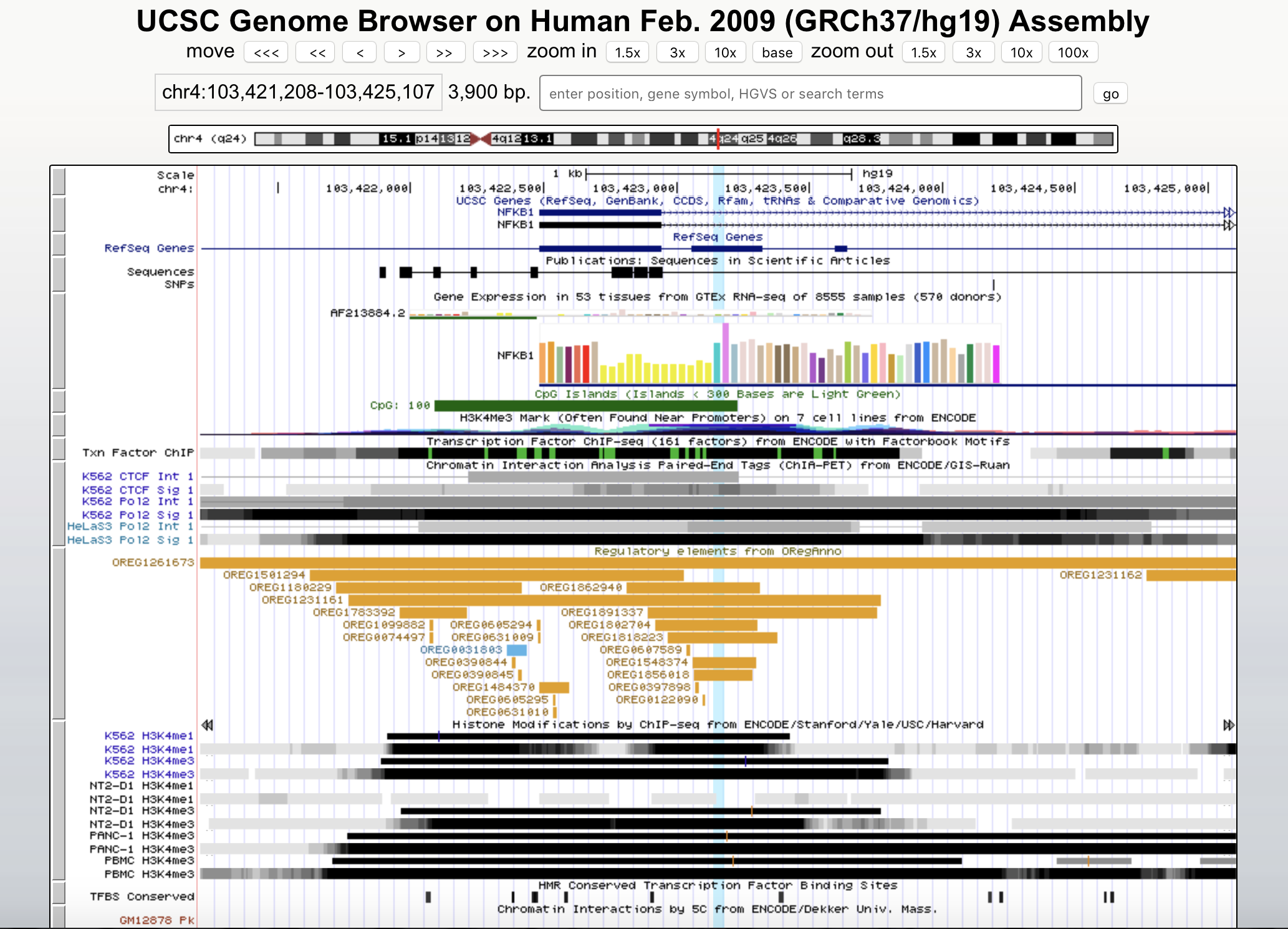


**Supplementary Figure 2.** *NFκB1* promoter overview generated by UCSC genome Browser (https://genome.ucsc.edu). The sequence analyzed is highlighted in light blue, showing that is in the promoter region of *NFκB1* and inside a CpG island and several transcription factor binding sites (*SMARCA4*, *SPL1*, *STAT1*, *RBL2*, *RB1* and *ETS1*) all of them determined by experimental procedures (ENCODE project).
